# Current Marijuana Use by Industry and Occupation — Colorado, 2014–2015

**DOI:** 10.15585/mmwr.mm6714a1

**Published:** 2018-04-13

**Authors:** Roberta Smith, Katelyn E. Hall, Paul Etkind, Mike Van Dyke

**Affiliations:** ^1^Colorado Department of Public Health and Environment; ^2^Private public health consultant.

The effects of marijuana use on workplace safety are of concern for public health and workplace safety professionals. Twenty-nine states and the District of Columbia have enacted laws legalizing marijuana at the state level for recreational and/or medical purposes. Employers and safety professionals in states where marijuana use is legal have expressed concerns about potential increases in occupational injuries, such as on-the-job motor vehicle crashes, related to employee impairment. Data published in 2017 by the Colorado Department of Public Health and Environment (CDPHE) showed that more than one in eight adult state residents aged ≥18 years currently used marijuana in 2014 (13.6%) and 2015 (13.4%) (*1*). To examine current marijuana use by working adults and the industries and occupations in which they are employed, CDPHE analyzed data from the state’s Behavioral Risk Factor Surveillance System (BRFSS) regarding current marijuana use (at least 1 day during the preceding 30 days) among 10,169 persons who responded to the current marijuana use question. During 2014 and 2015, 14.6% of these 10,169 Colorado workers reported current marijuana use, with the highest reported prevalence among workers in the Accommodation and Food Services industry (30.1%) and Food Preparation and Serving (32.2%) occupations. Understanding the industries and occupations of adults with reported marijuana use can help direct and maximize impact of public health messaging and potential safety interventions for adults.

The Colorado BRFSS is a CDC-sponsored, state-based, random-digit–dialed telephone survey of the noninstitutionalized U.S. population aged ≥18 years. The survey collects information on health risk factors, preventive health practices, and disease status (*2*). In 2012, 2014, and 2015, two standardized employment questions were included in the Colorado BRFSS survey (*3*). Respondents who indicated that their current employment status was employed for wages, self-employed, or out of work for less than 1 year were asked 1) “What kind of business or industry do you work in?” (industry classification), and 2) “What is your job title?” (occupation classification). In 2014 and in 2015, questions that collected information on marijuana use during the past 30 days were added to the Colorado survey. Respondents who replied “yes” when asked if they had ever used marijuana or hashish were then asked how many days during the past 30 days they had used marijuana or hashish as well as subsequent questions on use frequency and methods. Current use of marijuana was defined as having used marijuana or hashish on at least 1 day in the past 30 days.

Using the 2014 and 2015 BRFSS data combined, state-weighted percentages were calculated, and bivariate analyses using a Rao-Scott chi-square test were performed to compare the prevalence of marijuana use by age group, sex, and race/ethnicity. In addition, prevalence and 95% confidence intervals (CIs) were calculated to compare the prevalence of marijuana use by industry and occupation. Overall BRFSS industry and occupation data, representing current Colorado employment, were added to illustrate the percentage of employees working in the industries and occupations identified within the state. Percentages of employed persons reporting current marijuana use in the industry and occupation comparisons were age-adjusted based on the 2000 U.S. standard population. The overall response rate for the Colorado BRFSS was 57.0% in 2014 and 55.2% in 2015.

Among the combined 26,936 respondents* in the BRFSS 2014 and 2015 surveys, 18,848 (70.0%) were given the opportunity to answer the question of whether they had ever used marijuana or hashish, and 18,674 (99.1%) responded (either positively or negatively) to the question. Of those respondents, 10,169 (54.5%) indicated that they were employed or had been out of work for less than 1 year. Among the 10,169 workers responding, 14.6% reported using marijuana during the preceding 30 days (Table 1). The prevalence of current marijuana use was higher among persons aged 18–25 years (29.6%) than among persons aged 26–34 years (18.6%) and persons aged ≥35 years (11.0%), and higher among men (17.2%) than among women (11.3%). By race/ethnicity, prevalence of current marijuana use was highest among non-Hispanic whites (15.3%), followed by Hispanics (15.1%) and non-Hispanic blacks (14.5%) (Table 1).

**TABLE 1:** Self-reported current marijuana use among eligible employed adults (N = 10,169*), by selected characteristics — Behavioral Risk Factor Surveillance System, Colorado, 2014 and 2015

Characteristic	No.^†^	Current marijuana use % (95% CI)	p-value^§^
**Total**	**10,169**	**14.6 (13.6–15.7)**	**—**
**Age group (yrs)**			
18–25	625	29.6 (24.9–34.2)	<0.001
26–34	1,251	18.6 (15.7–21.4)	
≥35	8,187	11 (10–12)	
**Sex**			
Men	5,138	17.2 (15.7–18.7)	<0.001
Women	5,031	11.3 (9.9–12.8)	
**Race/Ethnicity**			
White, non-Hispanic	7,823	15.3 (14–16.5)	0.025
Black, non-Hispanic	259	14.5 (9–20)	
Other, non-Hispanic	194	5.7 (1.6–9.8)	
Multiracial, non-Hispanic	1,416	12.7 (10.2–15.3)	
Hispanic	270	15.1 (9.1–21.1)	

**Abbreviation:** CI = confidence interval.

* Respondents who indicated that they were employed or had been out of work for less than 1 year and who responded to the question of ever using marijuana or hashish.

**^†^** Age group missing for 106 (1.0%) respondents; race/ethnicity missing for 207 (2.0%).

**^§^** By Rao-Scott chi-square test.

Among the 10,169 workers, the industry with the highest prevalence of current marijuana use (30.1%) was Accommodation and Food Services (Table 2). Among occupations, Food Preparation and Serving had the highest prevalence of current marijuana users (32.2%), although the age-adjusted prevalence was 19.1% (Table 3).

**TABLE 2:** Prevalence of current marijuana use among eligible employed adults (N = 10,169*), ranked by industry, and overall percentage of state workers employed in the industry — Behavioral Risk Factor Surveillance System, Colorado, 2014 and 2015

Industry	Prevalence of current marijuana use, % (95% CI)	Age-adjusted prevalence of current marijuana use, % (95% CI)	Overall percentage of workers employed in the industry, % (95% CI)
Accommodation and Food Services	30.1 (23.4–36.7)	25.6 (17.3–34.0)	6.4 (5.7–7.1)
Arts, Entertainment, and Recreation	28.3 (19–37.6)	14.8 (7.7–22.0)	2.3 (1.9–2.8)
Other Services (except Public Administration)	20.9 (15.4–26.4)	21.2 (13.2–29.2)	4.3 (3.8–4.8)
Construction^†^	19.7 (16–23.4)	15 (10.4–19.6)	11.3 (10.4–12.2)
Real Estate, Rent, Lease	19.6 (13.6–25.7)	18.6 (9.1–28.0)	2.8 (2.4–3.2)
Retail Trade	18.9 (14.4–23.5)	18.0 (12.8–23.3)	9.4 (8.6–10.2)
Administration, Support, Waste Management, and Remediation Services	18.8 (13–24.7)	22.4 (14.4–30.5)	4.0 (3.4–4.5)
Information	18.2 (11.7–24.8)	18.1 (9.2–26.9)	3.2 (2.7–3.6)
Manufacturing^†^	16.3 (12–20.5)	17.3 (11.3–23.3)	6.9 (6.2–7.6)
Agriculture, Forestry, Fishing/Hunting^†^	14.4 (6.8–21.9)	18.3 (5.7–30.9)	2.1 (1.8–2.5)
Professional, Scientific, Technical Services	14.0 (10.4–17.7)	14.2 (8.7–19.8)	6.4 (5.8–7)
Finance and Insurance	13.5 (9–18.1)	8.9 (4.4–13.4)	4.0 (3.5–4.4)
Management of Companies and Enterprises	13.1 (0.0–30.3)	^—§^	0.2 (0.1–0.3)
Wholesale Trade	11.4 (4.8–17.9)	12.5 (3.4–21.7)	1.7 (1.3–2)
Transport and Warehousing^†^	10.2 (6–14.4)	10.7 (3.9–17.4)	4 (3.5–4.5)
Health Care and Social Assistance^†^	7.4 (5.5–9.4)	7.4 (4.3–10.5)	12.8 (11.9–13.6)
Education	5.8 (3.5–8.1)	6.1 (3.1–9.1)	7.4 (6.8–8.1)
Public Administration	5.8 (3.4–8.2)	5.6 (0.6–10.6)	7.1 (6.4–7.8)
Utilities^†^	5.8 (0.6–11.1)	3.2 (0.0–8.9)	1.4 (1–1.7)
Mining, Oil, and Gas^†^	5.2 (1.6–8.7)	6.9 (1.1–12.7)	2.3 (1.9–2.7)

**Abbreviation:** CI = confidence interval.

* Respondents who indicated that they were employed or had been out of work for less than 1 year and who responded to the question of ever using marijuana or hashish.

^†^ Industries that typically perform routine employee drug testing.

^§^ Not computed because of limited sample size.

**TABLE 3:** Prevalence of current marijuana use among eligible employed adults (N = 10,169*), ranked by occupation, and overall percentage of state workers employed in the occupation — Behavioral Risk Factor Surveillance System, Colorado, 2014 and 2015

Occupation	Prevalence of current marijuana use, % (95% CI)	Age-adjusted prevalence of current marijuana use, % (95% CI)	Overall percentage of workers employed in the occupation, % (95% CI)
Food Preparation and Serving	32.2 (23.8–40.5)	19.1 (11.9–26.3)	4.5 (3.9–5.2)
Arts, Design, Entertainment, Sports and Media	27.5 (19.6–35.3)	25.3 (16.5–34.0)	2.2 (1.8–2.5)
Production	20.8 (14–27.6)	21.3 (13–29.5)	3.8 (3.2–4.3)
Life, Physical, and Social Science	20.6 (12–29.3)	22.7 (10.6–34.8)	1.7 (1.4–2)
Sales and Related	19.4 (15–23.7)	19.1 (14–24.2)	10.0 (9.1–10.8)
Installation, Maintenance, and Repair	19.2 (12.3–26.1)	20.3 (8.3–32.3)	3.0 (2.5–3.5)
Personal Care and Service	16.8 (11–22.7)	16.6 (8.7–24.5)	3.4 (3–3.9)
Farming, Fishing, and Forestry^†^	16.5 (1.5–31.4)	17.3 (0.0–36.2)	0.7 (0.5–1)
Construction and Extraction^†^	16.5 (12.6–20.4)	12.2 (8–16.4)	9.1 (8.2–10)
Building and Grounds Cleaning and Maintenance	16.0 (10.6–21.4)	17.0 (9.8–24.3)	4.6 (4–5.2)
Legal	15.9 (8.5–23.3)	10.0 (0.0–20.1)	1.3 (1.1–1.6)
Healthcare Support^†^	15.8 (8.3–23.3)	15.5 (7.5–23.6)	2.4 (1.9–2.8)
Management	15.2 (12.2–18.2)	17.9 (11.7–24.1)	12.3 (11.4–13.1)
Computer and Mathematical	13.2 (7.8–18.6)	19.1 (7.7–30.4)	4.2 (3.7–4.8)
Office and Administrative Support	12.7 (9.9–15.5)	13.9 (9.1–18.7)	9.7 (9–10.5)
Architecture and Engineering	11.1 (6.3–15.9)	11.7 (3.4–20.0)	3.1 (2.6–3.5)
Business and Financial Operations	10.4 (6.8–14.1)	7.6 (3.3–12)	4.5 (4–4.9)
Transportation and Material Moving^†,§^	10.3 (6.1–14.4)	10.4 (3.2–17.7)	5.2 (4.6–5.8)
Community and Social Services	6.7 (1.9–11.5)	7.6 (0.0–16.4)	1.2 (1–1.5)
Education, Training, and Library	6.3 (3.2–9.5)	6.8 (2.9–10.7)	5.1 (4.6–5.6)
Protective Service	6.2 (0.8–11.6)	0.1 (0.0–0.4)	2.3 (1.9–2.7)
Healthcare and Technical^†^	3.1 (1.5–4.8)	1.9 (0.0–3.7)	5.6 (5–6.1)

**Abbreviation:** CI = confidence interval.

* Respondents who indicated that they were employed or had been out of work for less than 1 year and who responded to the question of ever using marijuana or hashish.

^†^ Safety-sensitive occupations in which workers have responsibility for their own safety or the safety of others.

^§^ Subject to federal drug testing requirements.

Among safety-sensitive occupations (those in which workers have responsibility for their own safety or the safety of others), prevalences of current marijuana use among workers who acknowledged using marijuana or hashish in the preceding 30 days and were employed in Construction and Extraction (16.5%); Farming, Fishing, and Forestry (16.5%); and Healthcare Support (15.8%) were higher than the overall state prevalence of 14.6% among employed adults. However, the prevalences of current marijuana use among workers in Transportation and Material Moving (10.3%) and Healthcare and Technical (3.1%) were lower than the overall state prevalence (Table 3)

Reported current use of marijuana was lower in industries that are known to perform routine drug testing on employees such as the Healthcare and Social Assistance (7.4%); Utilities (5.8%); and Mining, Oil, and Gas industries (5.2%) (Table 2). Current use also was lower than the overall state prevalence in Transportation and Material Moving occupations (10.2%), which are subject to federal drug testing requirement (Table 3).

## Discussion

This is the first study to use BRFSS data to describe self-reported current marijuana use among adults working in various industries and occupations. Although reported past-month marijuana use does not necessarily indicate use or impairment on the job, there is some evidence that marijuana use in general might increase the risk for nondriving workplace injuries (*4*) Motor vehicle crashes are the leading cause of work-related deaths in the United States (*5*), and studies have linked recent marijuana use to an increased risk for motor vehicle crashes (*6*,*7*). However, although marijuana negatively affects skills needed for safe driving, limitations related to roadside and toxicology testing, marijuana detection time, and co-use of substances contribute to uncertainty about risk. A 2006 study using 2000–2001 data from the National Household Surveys on Drug Abuse and the 2002 National Survey on Drug Use and Health found that workers who were subject to frequent workplace drug testing and severe penalties were less likely to report past month marijuana use (*8*). Because BRFSS is a public health survey, reporting marijuana use might be more representative of actual use by industry and occupation than if this information had been collected through an employer-sponsored survey.

Analysis of age-adjusted prevalences among workers who acknowledged ever using marijuana or hashish highlighted the impact of younger workers in various industries and occupations on prevalence rates. In industries that tend to attract younger workers, such as food services, the marijuana use prevalence decreased with age-adjustment. For example, whereas the unadjusted prevalence of marijuana use among adults employed in Food Preparation and Serving occupations was 32.2%, the age-adjusted prevalence was 19.1%. Similarly, there were large differences in adjusted and unadjusted prevalences in the Arts, Entertainment, and Recreation industry, which might have a large proportion of younger workers.

The findings in this report are subject to at least six limitations. First, data were collected from adults in Colorado who reported being employed at the time of the survey and might not be representative of all employed adults in Colorado. Second, among respondents to the marijuana question, not all responded to the question regarding which industry or occupation they were employed in or recorded an industry or occupation that could be coded to an existing industry or occupation. This resulted in missing data for the 10,169 workers analyzed. Third, industry and occupation information were reported by respondents who were currently employed for wages, self-employed, or out of work for less than 1 year. A respondent might not have been actively working in the industry or occupation recorded, and that could influence the prevalence of marijuana use. Fourth, data are self-reported and thus are subject to the limitations for such survey data, including recall and response bias. Fifth, self-reported data might be subject to interviewer and recording errors leading to misclassification. These estimates might differ from other nationally representative behavior surveillance systems because of differences in survey methods, survey type, and topic. Finally, current use of marijuana was defined as having used marijuana or hashish on at least 1 day in the past 30 days. An employee who uses marijuana every day versus one that uses only once a month might present different considerations for impairment in a workplace. It is also important to note that these data do not directly measure working under the influence of marijuana.

This analysis provides important data for employers considering or implementing workplace marijuana policies and highlight those industries where marijuana use among workers might reflect a higher proportion of younger workers, such as Accommodation and Food Services and Arts, Entertainment, and Recreation. Awareness of possible employee recreational marijuana use can inform employer policies regarding drug use and workplace impairment. For example, safety-sensitive industries that have higher prevalences of self-reported marijuana use could consider evaluating their current drug testing programs, drug panels used for preemployment screening, and testing frequencies, and develop policies regarding tolerance of drug use. Drug testing policies need to be explained clearly, including expectations around protocols when injuries occur. Age-adjusted employment data can help to potentially target responsible use education campaigns to particular occupations and industries that employ younger workers.

**Summary**

**What is already known about this topic?**

Eight states, including Colorado, have legalized recreational marijuana use among persons aged ≥21 years. The association between marijuana use and occupational injury is of public health concern.

**What is added by this report?**

During 2014–2015, 14.6% of 10,169 Colorado adult workers reported using marijuana in the past 30 days. The highest prevalences of current use were among young adults and men, and among adults working in the Accommodation and Food Services industry (30.1%) and Food Preparation and Serving occupation (32.2%).

**What are the implications for public health practice?**

By understanding the occupations and industries of workers who report recreational marijuana use, employers can develop appropriately targeted workplace marijuana policies and safety awareness campaigns.

## Footnotes

* Included adults who were students, retirees, and homemakers, in addition to those employed.
